# The relationship between polymorphisms in the promoter region of Tim-3 and unexplained recurrent spontaneous abortion in Han Chinese women

**DOI:** 10.1186/1477-7827-11-104

**Published:** 2013-11-11

**Authors:** Yang Shen, Chen Wang, Dun Hong, Baojin Zeng, Congcheng Fang, Chiting Yuan, Lilong Fan, Haiyan Lv, Min Zhu

**Affiliations:** 1 The Public Laboratory, Taizhou Hospital of Zhejiang Province, Wenzhou Medical College, Linhai, Zhejiang 317000, China; 2 The department of obstetrics and gynaecology, Taizhou Hospital of Zhejiang Province, Wenzhou Medical College, Linhai, Zhejiang 317000, China

**Keywords:** Unexplained recurrent spontaneous abortion, *Tim-3*, Gene polymorphism, Cytokines

## Abstract

**Background:**

Recurrent spontaneous abortion (RSA) refers to 2 or more consecutive pregnancy losses, and RSA with unknown causes is called unexplained recurrent spontaneous abortion (URSA). Tim-3, a subtype of the T-cell immunoglobulin domain and mucin domain (Tim) protein family, might be an important regulatory molecule that plays a pivotal role in URSA, which might be triggered mostly by Th1/Th2 immune deviation. To understand the etiology and pathogenesis of URSA in Han Chinese women, we investigated the association between polymorphisms of rs10053538 and rs10515746 in the promoter of *Tim-3* and the risk of URSA in Han Chinese women.

**Methods:**

One hundred and forty-eight women with RSA resulting in still birth were enrolled in the URSA group. We performed tests to rule out congenital reproductive system malformation, reproductive system tumor, endocrine dyscrasia, and chromosome abnormalities. One hundred and fifty-three women with normal pregnancy leading to live birth were selected at random to comprise the control group. All women included in this study were genetically unrelated Han Chinese women. Polymerase chain reaction-restriction fragment length polymorphism (PCR-RFLP) and allele-specific polymerase chain reaction (AS-PCR) were used to determine polymorphisms of rs10053538 and rs10515746, respectively, in all subjects. PCR products were chosen at random for sequencing.

**Results:**

No significant statistical difference was found between the distribution frequency of the GT + TT genotype and T allele on the rs10053538 locus in the URSA group or the control group (10.1% vs. 11.8%, Chi(2) = 0.205, *P* = 0.651; 5.1% vs. 6.5%, Chi(2) = 0.592, *P* = 0.441; respectively). Neither was there a significant difference between the distribution frequency of the GT + TT genotype and T allele on the rs10515746 locus in the groups (6.8% vs. 3.9%, Chi(2)1.201, *P* = 0.273; 3.4% vs. 2.0%, Chi(2) = 1.169, *P* = 0.280; respectively).

**Conclusions:**

The present study suggested that these polymorphisms of rs10053538 or rs10515746 in the *Tim-3* promoter may not be associated with URSA in Han Chinese women.

## Background

Recurrent spontaneous abortion (RSA) refers to 2 or more consecutive pregnancy losses [1]. RSA is pervasive in clinical pathological pregnancy, but its pathogenesis is very complicated. Besides chromosome abnormalities, anatomic abnormality, endocrine dyscrasia, and infection, the etiology and pathogenesis of 40–60% RSA patients are not understood. RSA with unclear causes is called unexplained recurrent spontaneous abortion (URSA) [2].

From the view of immunology, a normal pregnancy can be considered a type of successful HLA-haploidentical allogeneic transplant. In a normal pregnancy, the mother’s immune tolerance against the father’s alloantigens is very strong, and the embryo can be comparable to an allograft of semi-identical HLA [3]. It has been reported that special Th2-cell - related phenomena occur in the mother in a normal pregnancy. The Th1 cells are suppressed, and the Th1-type cytokines (such as IL-2, IFN-γ, and TNF-α) are present at an extremely low level or may even be absent. Th2 cells are mainly assigned to mediate humoral immune response, stimulate B-cells to secrete antibodies, and maintain allograft immune tolerance. Th2-type cytokines contribute greatly to a normal pregnancy [4]. A disequilibrium of the Th1/Th2 balance together with the over-functioning of Th1-cells leads to susceptibility to pathological pregnancy, including abnormal implantation, fetal growth retardation, fetal growth arrest, and URSA [5,6].

Tim-3, a subtype of the T-cell immunoglobulin domain and mucin domain (Tim) protein family, which was first identified in 2001 [7], is selectively expressed on Th1 cells but not on Th2 cells and can induce the apoptosis of Th1 cells after specific binding with its main ligand galectin-9 [8]. Popovici et al. found that the expression of galectin-9 was much higher in early decidual cells than in non-pregnant endometrium epithelial cells [9]. Thus,it is likely that allograft tolerance weakens after the Tim/galectin-9 pathway is blocked, for the dismissal of Th1 cell suppression and the enhanced function of Th1 cells, including the upregulated expression of Th1 cytokines and Th1-type immune response overbalance. Inversely, activating the Tim-3/galectin-9 pathway would suppress the immune response of Th1 cells and benefit the immune tolerance of allografts [5]. Moreover, Tim-3 expressed on natural killer (NK) cells was found to be elevated when they were activated, suppressing the NK-dependent immune response and strengthening immune tolerance [10].

*Tim-3* polymorphism has also been shown to change the interaction between Tim-3 and its ligand, thereby affecting the processes leading to some immune diseases [11]. Given all these findings, Tim-3 might be an important regulatory molecule that plays a pivotal role in URSA, which might be triggered mostly by Th1/Th2 immune deviation. Whether anomalies in the activation of the Tim-3/gelatin-9 pathway, for example, change in the structure of Tim-3/gelatin-9, their abnormal expression, or mutual interaction lead to URSA, remains unknown. *Tim-3/Tim-3* polymorphisms and their association with URSA in Han Chinese women have not been studied thus far. Here, we examined polymorphisms of rs10053538/rs10515746 loci in the promoter of Tim-3 and their relevance to URSA in Han Chinese women.

## Methods

The protocols of this research were approved by the Ethics Committee of Taizhou Hospital. Written informed consent was obtained from all patients engaged in this study at the start of the research. Because all the researchers of this study work for Taizhou Hospital, which is located in Taizhou, Zhejiang, a Han Chinese habitation in China, all subjects selected were Han Chinese women.

### Patients and DNA samples

One hundred and forty-eight women with RSA resulting in still birth were enrolled from the Obstetrics and Gynecology Department of Taizhou Hospital to comprise the research group. In addition, 153 patients with normal pregnancy culminating in live birth were selected at random from the Obstetrics and Gynecology Department of Taizhou Hospital to form the control group. They had delivered at least 1 full-term healthy baby without the aid of assisted reproductive technologies and had not experienced miscarriage or pregnancy complications in the past. The abortions included both embryonic and anembryonic losses before 20 weeks of gestational age, which were determined by ultrasound dating and/or dating based on the last menstrual period. All subjects had undergone a comprehensive examination, including determination of detailed medical history, physical examination, beta subunit of human chorionic gonadotropin (BhCG), and transvaginal three-dimensional ultrasonography. They were also tested for 75 g oral glucose tolerance, thyroid functions, anti-cardiolipin antibodies (IgG/IgM), lupus anticoagulant, anti-thrombin III, protein S, and protein C to rule out glucose intolerance, endocrine dyscrasia, thrombophilia factors, urogenital tumor, and congenital reproductive system malformation. Patients with chromosome abnormalities were also excluded from the study. All patients included in this study were genetically unrelated Han Chinese women. The genomic DNA of white blood cells from every sample was extracted according to the recommended procedure of the DNA isolation kit (Generay, SH, China). The extracted genomic DNA was dissolved in sterile double disstilled water. The concentrations and A260/A280 ratios of the DNA solutions were measured using a nucleic acid spectrometer (Bio-Rad, CA, USA). DNA samples with A260/280 ratios ranging from 1.7 to 2.0 were chosen as polymerase chain reaction (PCR) templates and were preserved at -80°C.

### rs10515746 genotyping

Allele-specific polymerase chain reaction (AS-PCR) was used to examine the rs10515746 locus, and 3 primers were designed according to the flanking sequence [GenBank: NM_032782]. The primer sequences were as follows: F1, 5′-GGCTTATGCTGGGAGTTGCT-3′; F2, 5′-GGCTTATGCTGGGAGTTGCG-3′; and R1, 5′-GGTGTCTGATTGCCAGTGATTC-3′. F1 and R1 were used to amplify T allele fragments, and F2 and R1 were used to amplify G allele fragments; all the amplified fragments were 499 bp long. Every sample was subjected to PCR with F1/R1 and F2/R1. The reaction mixture included 10× PCR buffer, 2.5 μL; dNTP (10 mM each), 0.5 μL; forward primer, 0.25 μL (20 mmol/L); reverse primer, 0.25 μL (20 mmol/L); DNA template, 0.1 μg; and *Taq* DNA polymerase, 2 U. The final volume of the mixture was made up to 25 μL with double distilled water. Touchdown PCR was used to amplify the target amplicons. The reaction was heated on a Thermo Cycler (Bio-rad, CA, USA) to 95°C for 2 min, followed by 94°C for 30 s, annealing temperature is progressively lowered from 62°C to 54°C by 1°C every 2 cycles, annealing for 30 s in every cycle, extension for 40 s, followed by 12 additional cycles at 54°C for 40 s, 28 cycles were conducted totally, final extension at 72°C for 10 min. The amplified products were subjected to 1.8% agarose gel electrophoresis, visualized by ethidium bromide (EB) staining, and photographed by FireReader (UVItec, CB, UK).

### rs10053538 genotyping

Primers were designed according to the flanking sequence of rs10053538 provided by GenBank [GenBank: NM_032782]. Sequences of forward and reverse Tim-3 primers were as follows: 5′-ATGGTCATTTGCTGGTTATTAGTT-3′ and 5′-ATGTTGGTCAGGCTGTTCTT-3′. The PCR mix components were as follows: 10× PCR buffer, 2.5 μL; dNTP (10 mM each), 0.5 μL; forward primer, 0.25 μL (20 mmol/L); reverse primer, 0.25 μL (20 mmol/L); DNA template, 0.1 μg; and *Taq* DNA polymerase, 2 U. The final volume was made up to 25 μL with double distilled water. The reaction mixture was subjected to PCR in a thermal cycler (Bio-Rad) under the following conditions: 95°C for 2 min; followed by 35 cycles each of 94°C for 45 s, 59°C for 45 s, 72°C for 1 min; and a final extension step of 72°C for 10 min. Next, 5 μL of the amplified products was used for 1.5% agarose gel electrophoresis. The PCR products were visualized by ethidium bromide (EB) staining and photographed by FireReader. DNA digestion conditions were as follows: PCR products, 1 μg; 10× buffer solution, 2.0 μL; and restriction endonuclease *Bse*LI, 2.5 U. The final volume was made up to 20 μL with double distilled water. PCR products were digested for 16 h at 55°C. The digestion products were subjected to 2.0% agarose gel electrophoresis, visualized by EB staining, and photographed by FireReader.

### DNA sequencing

Some of the PCR products were randomly selected and then purified with a PCR purification kit. The purified products were sent to GeneCore BioTechnologies Co. Ltd., Shanghai for DNA sequencing.

### Statistical analysis

Statistical analysis was performed using the SPSS 18.0 software package. The weight factors of both the wild-type homozygote GG and mutant-type homozygote TT were 2, while the weight factor of mutant heterozygote GT was 1. The allele frequencies (constituent ratio) of each group were calculated. G(T) allele frequency = (weight factor of homozygote × the number of homozygote GG(TT) + weight factor of homozygote × the number of heterozygote GT)/(2 × the number of patients being studied). The χ^2^ test was adopted to compare the allele frequencies of each group. Hardy-Weinberg equilibrium test was adopted to examine the demographic representation of the research group and control group. The relative risks were presented as the odds ratios (OR) and their 95% confidential intervals (CI). Significance was defined as *P* < 0.05.

## Results

### General characteristics

The ages of the URSA patients ranged from 22 to 44 years old, while the ages of the control subjects ranged from 21 to 40 years old. The mean ages of the research group and control group were 29.8 ± 4.3 years old and 28.5 ± 4.0 years old, respectively. There was no statistically significant difference between the ages of the 2 patient groups (*P* > 0.05). The χ^2^ values of Hardy-Weinberg equilibrium test of rs10053538/rs10515746 locus in the URSA group were 0.42 (*P* = 0.52)/0.18 (*P* = 0.67), while χ^2^ values of the control group were 3.18 (*P* = 0.07)/0.06 (*P* = 0.80), respectively. These results suggested that the 2 groups in our study were in Hardy-Weinberg equilibrium and were demographically representative.

### rs10515746

**Figure 1:**
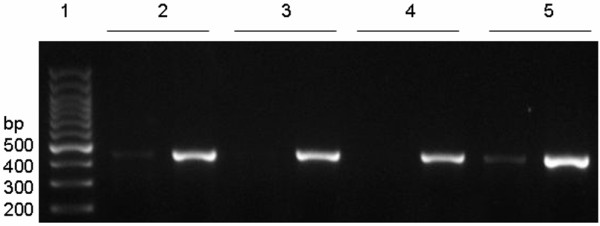
**Electrophoretogram of rs10515746 locus in Tim-3 gene.** (1) represented 100 bp DNA marker; (2, 5) represented genotype of GT; (3, 4) represented genotype of GG.

**Figure 2:**
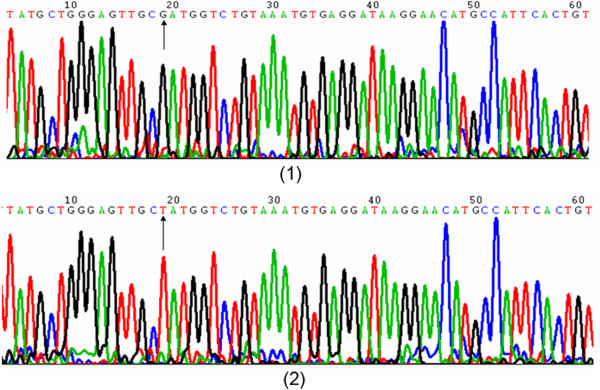
**Sequence diagrams of rs10515746 locus in Tim-3 gene.** (1) represented G allele; (2, 5) represented T allele (heterozygote).

At this locus, G and T alleles were defined as wild-type and mutant alleles, respectively. After agarose gel electrophoresis, the GG genotypes were defined as wild GG homozygotes, TT genotypes as mutant TT homozygotes, and GT genotypes as GT heterozygotes (Figure 1). In the present study, 138 URSA patients had the GG genotype, and the other 10 URSA patients had the GT genotype. However, 147 control subjects had the GG genotype, and the other 6 had the GT genotype. No patient in the URSA group or the control group had the TT genotype (Figure 2).

### rs10053538

**Figure 3:**
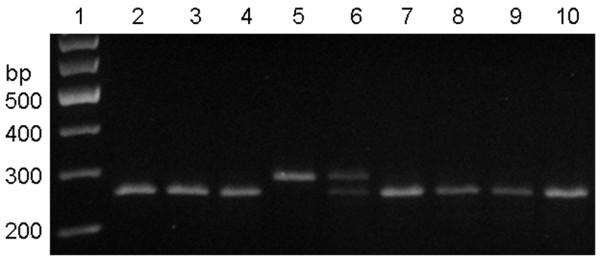
**Electrophoretogram of post-digestion products of rs10053538 locus in Tim-3 gene.** (1) represented 100 bp DNA marker; (2, 3, 4, 7, 10) represented genotype of GG; (5) represented genotype of TT, (6) represented genotype of GT.

**Figure 4:**
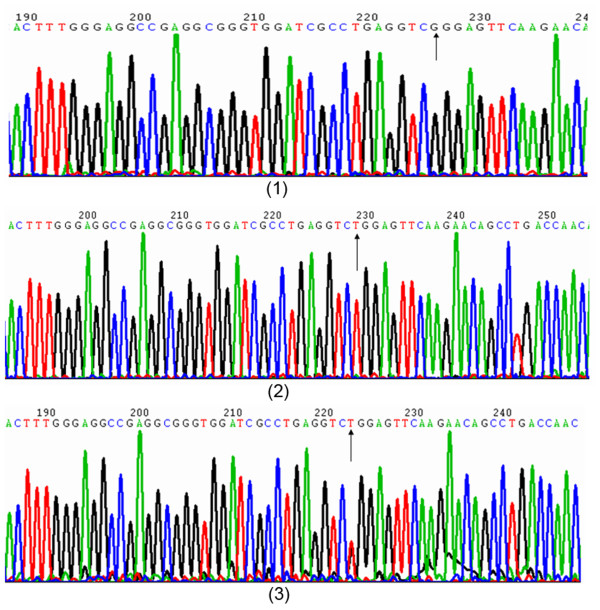
**Sequence diagrams of rs10053538 locus in Tim-3 gene.** (1) represented G allele, (2) represented T allele (homozygote), (3) represented genotype of GT (heterozygote).

At the rs10053538 locus, the genetic polymorphism was demonstrated as 3 genotypes (GG, GT, and TT). Because PCR products of the GG genotype possessed a recognition site of *Bse*LI endonuclease, they could be cleaved into 2 DNA fragments, namely, of 252 bp and 30 bp, after digestion. Mutation of this locus (from G to T) disrupts the recognition site of *Bse*LI endonuclease. Hence, the PCR products of the TT genotype could only give rise to 1 kind of fragment, i.e., of 282 bp, while the PCR products of GT genotype could be split into 3 kinds of DNA fragments: 282 bp, 252 bp, and 30 bp. Unfortunately, due to their low molecular weight, the 30 bp DNA fragments could not be classified by agarose gel electrophoresis (Figure 3). Here, 133 URSA patients had the GG genotype, and the other 15 URSA patients had the GT genotype; meanwhile, in the control group, 135 had the GG genotype, 16 had the GT genotype, and 2 had the TT genotype (Figure 4).

### Genotypes and frequencies

At the rs10053538 locus on *Tim-3*, the GG and (GT + TT) genotypes had frequencies of 89.9% and 10.1%, respectively, in the URSA group. In contrast, 88.2% of the control subjects had the GG genotype, and the other 11.8% had the (GT + TT) genotype. There was no significant statistical difference between the 2 groups (χ^2^ = 0.205, *P* = 0.651). At the rs10515746 locus on *Tim-3*, the genotype frequencies for GG and (GT + TT) were 93.2% and 6.8%, respectively, in the URSA group. In the control group, 96.1% of the subjects had the GG genotype, and the other 3.9% had the (GT + TT) genotype. There was no significant statistical difference between the different genotype frequencies in the 2 groups (χ^2^ = 1.201, *P* = 0.273). The frequency of GT + TT on rs10053538/rs10515746 loci in the URSA group was of not statistically different from that of the control group (P = 0.651, OR = 1.182; *P* = 0.273, OR = 0.563) (see Table 1). The frequencies of T allele on rs10053538/rs10515746 loci in the 2 groups were not statistically different either (*P* = 0.441, OR = 1.310; *P* = 0.280, OR = 0.572) (see Table 2).

**Table 1:** Distribution of Tim-3 genotypes of the studied population(%)

**Genotype**	**URSA**	**Control**	***χ***^***2***^	***P***	***OR***	**95% *CI***
	**(n = 148)**	**(n = 153)**				
*rs10053538*						
*GG*	133(89.9)	135(88.2)	0.205	0.651	1.182	0.572-2.443
*GT + TT*	15 + 0(10.1)	16 + 2(11.8)				
*rs10515746*						
*GG*	138(93.2)	147(96.1)	1.201	0.273	0.563	0.199-1.591
*GT + TT*	10 + 0(6.8)	6 + 0(3.9)				

**Table 2:** Distribution of different alleles on rs10053538 and rs10515746 (%)

**Alleles**	**URSA**	**Control**	***χ***^***2***^	***P***	***OR***	**95% *CI***
	**(n = 148)**	**(n = 153)**				
*rs10053538*						
*G*	281(94.9)	286(93.5)	0.592	0.441	1.310	0.657-2.610
*T*	15(5.1)	20(6.5)				
*rs10515746*						
*G*	286(96.6)	300(98.0)	1.169	0.280	0.572	0.205-1.594
*T*	10(3.4)	6(2.0)				

## Discussion

Tim-3, also known as hepatitis A virus cellular receptor 2 (HAVCR2), is a newly discovered surface molecule on T cells, with important immune regulatory functions. In humans, *Tim-3* is located on chromosome 5q33.2. Tim-3 comprises 301 amino acids, including elementary structural domains such as the signal peptide domain, immunoglobulin V (IgV) structural domain, mucin-like structural domain, transmembrane zone, and cytomere domain of phosphorylation site. In the IgV structural domain, there are 2 antiparallel β fragments and a metal ion ligand binding site (MILIBS), which together function as the ligand-binding site of Tim-3 [12,13].

In this study, rs10053538 and rs1055746 loci of *Tim-3* were studied. Statistical data showed that in both healthy women and URSA patients, the frequencies of mutant-type homozygote TT on both loci were low. Hence, the data of mutant-type homozygote TT and mutant-type heterozygote GT were merged and re-analyzed by χ^2^ test. However, still, no significant statistical difference was found between the distribution frequencies of GT + TT genotype in either group (Table 1). The distribution frequencies of T allele on rs10053538/rs10515746 locus of each group were of no statistical difference either (see Table 2). All these data suggested that the polymorphisms of rs10053538/rs10515746 may not contribute to the URSA in Han Chinese women.

Tim-3 deficiency has been reported to precipitate the aggravation of diseases in which the Th1-type immune response prevails, increasing the magnitude of illness and mortality [14]. Furthermore, the Th1/Th2-type cytokine balance leans toward a radical overbalance of Th1-type cytokine in URSA patients [4]. Herein, it can be postulated that *Tim-3/Tim-3* polymorphisms might be closely related to URSA.

Meanwhile, researchers found that the distribution frequencies of +4259 T/G in *Tim-3* in patients with pancreatic cancer or renal cell carcinoma were statistically different from those of healthy people [15,16]. The distribution frequencies of +4259 T/G in *Tim-3* in rheumatoid arthritis patients have been shown to be statistically different compared with those of healthy people both in the Han Chinese and Hui Chinese [17]. According to another report, in Korea, the distribution frequencies of +4259 T/G in *Tim-3* in children with dermatitis or asthma were not statistically different from those of healthy children, but this was not so in Caucasian or Hispanic children [18].

Given these findings, it might be concluded that *Tim-3/Tim-3* polymorphisms and their association with diseases not only pertain to the inclusion criteria of subjects but also to ethnic and regional differences.

Despite the current data don’t complying with the postulation, the polymorphisms of rs10053538/rs10515746 loci in *Tim-3* and their relationship with URSA are still not that clear. This is because of several reasons. First, this study included as research subjects only Han Chinese women in Taizhou, a Han Chinese habitation. Second, not all patients in this study had a precise record of miscarriage history. The number of miscarriages was self-reported; therefore, there might have been errors in the precise number of miscarriages. Third, many studies have found that Tim-3 plays an important role in certain immune diseases. Therefore, the polymorphisms in *Tim-3* and their association with URSA, such as the relationship between genotype and phenotype and their function in the molecular genetic mechanisms of URSA, warrant further investigation.

## Conclusions

The polymorphism of rs10053538/rs10515746 may not contribute to the URSA in Han Chinese women. However, investigation of other SNP sites of the *Tim-3* gene and their relationship with susceptibility to URSA might reveal meaningful associations.

## Abbreviations

AS-PCR: Allele-specific polymerase chain reaction; BhCG: Beta subunit of human chorionic gonadotropin; CI: Confidence intervals; EB: Ethidium bromide; HAVCR2: Hepatitis A virus cellular receptor 2; IgV: Immunoglobulin V; MILIBS: Metal ion ligand binding site; NK cells: Natural killer cells; OR: Odds ratios; PCR: Polymerase chain reaction; PCR-RFLP: Polymerase chain reaction-restriction fragment length polymorphism; RA: Rheumatoid arthritis; RSA: Recurrent spontaneous abortion; SNP: Single-nucleotide polymorphism; URSA: Unexplained recurrent spontaneous abortion.

## Competing interests

The authors declare that they have no competing interests.

## Authors’ contributions

YS carried out the molecular genetic studies and drafted the manuscript. CW, DH, and BZ collected all samples and performed the clinical tests. CF, CY, LF, and HLV helped in carrying out the molecular genetic studies and performed the statistical analysis. MZ participated in the study design and coordination and helped draft the manuscript. All authors have read and approved the final manuscript.
